# Utility of Different Adherence Measures for PrEP: Patterns and Incremental Value

**DOI:** 10.1007/s10461-017-1951-y

**Published:** 2017-10-31

**Authors:** Andrew Abaasa, Craig Hendrix, Monica Gandhi, Peter Anderson, Anatoli Kamali, Freddie Kibengo, Eduard J. Sanders, Gaudensia Mutua, Namandjé N. Bumpus, Frances Priddy, Jessica E. Haberer

**Affiliations:** 1 MRC/UVRI Uganda Research Unit on AIDS, P.O Box 49, Entebbe, Uganda; 2 Division of Clinical Pharmacology, Department of Medicine, Johns Hopkins University School of Medicine, Baltimore, ME USA; 3 Divisions of HIV, Infectious Diseases and Global Medicine, Department of Medicine, University of California, San Francisco, CA USA; 4 Department of Pharmaceutical Sciences, University of Colorado, Aurora, CO USA; 5 International AIDS Vaccine Initiative, New York, NY USA; 6 Kenya Medical Research Institute, University of Oxford, Kilifi, Kenya; 7 Kenya AIDS Vaccine Initiative, University of Nairobi, Nairobi, Kenya; 8 Massachusetts General Hospital Global Health and Harvard Medical School, Boston, MA USA

**Keywords:** PrEP drug-taking patterns of adherence electronic monitoring, Hair, Plasma

## Abstract

Measuring PrEP adherence remains challenging. In 2009–2010, the International AIDS Vaccine Initiative randomized phase II trial participants to daily tenofovir disoproxil fumarate/emtricitabine or placebo in Uganda and Kenya. Adherence was measured by electronic monitoring (EM), self-report (SR), and drug concentrations in plasma and hair. Each adherence measure was categorised as low, moderate, or high and also considered continuously; the incremental value of combining measures was determined. Forty-five participants were followed over 4 months. Discrimination for EM adherence by area under receiver operating curves (AROC) was poor for SR (0.53) and best for hair (AROC 0.85). When combining hair with plasma or hair with self-report, discrimination was improved (AROC > 0.9). Self-reported adherence was of low utility by itself. Hair level was the single best PK measure to predict EM-assessed adherence; the other measurements had lower discrimination values. Combining short-term (plasma) and long-term (hair) metrics could be useful to assess patterns of drug-taking in the context of PrEP.

## Introduction

According to UNAIDS, an estimated 2 million individuals acquired HIV in 2015 globally, with infection rates being highest in sub-Saharan Africa [1]. There is therefore an urgent need for effective methods to prevent ongoing transmission of HIV. A number of clinical trials examining the efficacy of oral pre-exposure prophylaxis (PrEP) [2–6] with tenofovir disoproxil fumarate/emtricitabine (TDF/FTC) have been published in recent years. Results from these studies have been largely positive, indicate that PrEP prevents acquisition of HIV infection when users are adherent [2, 4], and have led to broad recommendations for PrEP use worldwide [7].

Accurately measuring adherence to PrEP remains a challenge. In the HIV treatment setting, adherence is equally difficult to measure but viral loads can serve as a surrogate of adherence, unlike in the context of PrEP. Although multiple metrics of PrEP adherence have been investigated, there is no gold standard. Non-pharmacokinetic (non-PK) measures include self-report, pharmacy refills, pill counts, and electronic monitoring (EM; pill bottles that record each opening). Pharmacokinetic (PK) metrics are also available, such as monitoring drug levels in plasma, peripheral blood mononuclear cells (PBMCs), dried blood spots (DBS), or hair. Various combinations of these measures have been used in the PrEP trials conducted to date, and recent papers have found different degrees of concordance among them [8–13]. Self-report is generally found to over-estimate adherence and correlate poorly with adherence determined by objective measures, such as drug levels in plasma, EM, and pill count [5, 9, 10]. In the International AIDS Vaccine Initiative (IAVI) phase II trials of PrEP conducted among serodiscordant couples in Uganda [13] and men who have sex with men (MSM) and female sex workers (FSW) in Kenya [14], modest associations were observed between PK measures (plasma, PBMC, and scalp hair levels) and adherence assessed by EM, but there was little correlation between self-report and drug levels in any matrix.

Discrepant results with different adherence measures could imply bias and/or inaccuracy with one or more of the measures. For instance, social desirability bias and recall error commonly limit the utility of self-reported adherence. EM may be inaccurate if individuals remove more than one pill per opening or do not remove any pills with an opening. Moreover, removing pills from a container may not necessarily translate to drug ingestion. PK metrics are influenced by the half-life of the drug moiety and could be influenced by the timing of the sample draw relative to dosing (for short-term metrics), as well as biological and analytical variability in assay performance. Moreover, drug levels in different biomatrices such as hair and plasma measure adherence over different time periods and a patient may not be uniformly adherent over time. A significant limitation common to many comparisons of PK and non-PK measures is that the reporting periods are not always aligned. For example, a self-reporting period may be 1 month in duration and that period may be compared with a tenofovir concentration in plasma, which reflects short-term drug ingestion (1–7 days). Tenofovir drug levels in PBMCs reflect ingestion over the moderate-term (7–14 days) and levels in hair and dried blood spots reflect ingestion over the long term (weeks to months). Finally, analyses comparing adherence measures in PrEP trials to date have also not yet explored the incremental value of using multiple adherence measures, adjusting them to reflect the same duration of PrEP use. Guidance for adherence measurement in PrEP trials, demonstrations projects and real world roll-out is therefore needed.

In this analysis, we provide a comprehensive assessment of multiple non-PK and PK adherence measures adjusted to align over a set duration of time within a phase II PrEP trial in two distinct populations: MSM and serodiscordant couples. We use short and long-term PK measures to assess patterns and concordance of adherence to PrEP over time. We also determine the incremental value of assessing multiple adherence measures in combination (self-report and drug levels in plasma and hair) compared to EM.

## Methods

### Trial Setting and Participants

The phase II trial (conducted before the phase 3 trials showed efficacy of PrEP) was conducted at three of the IAVI-partner clinical research centres in Africa: the Medical Research Council/Uganda Virus Research Institute in Masaka, Uganda; the Kenya AIDS Vaccine Initiative in Kangemi, Nairobi, Kenya; and the KEMRI-Wellcome Trust Research Programme in Kilifi, Kenya. Eligible participants were HIV-negative adults aged 18–49 years. In Uganda, participants were HIV-uninfected partners in known HIV-serodiscordant couple relationships and had self-reported unprotected sex with one or more HIV-infected partners not taking antiretroviral therapy (ART) in the past 3 months. Participants in Kenya were HIV-uninfected MSM and HIV-uninfected FSW. Enrolment took place from October 2009 through March 2010. The details of study procedures have been described previously [13, 14]. Briefly, eligible participants were randomized to daily TDF/FTC or placebo or intermittent TDF/FTC (a fixed dose on Mondays and Fridays, and a post-coital dose within 2 h after sex, not to exceed one dose per day) or placebo in a 2:1:2:1 ratio. The trial objectives were to evaluate the safety and acceptability of daily and intermittent dosing of TDF/FTC, measure adherence to the two dosing strategies via an array of metrics, and evaluate changes in HIV-associated risk behaviour with the two dosing strategies. Participants were followed at Weeks 1, 2, and 4 post-enrolment and then monthly for 4 months with standardized adherence and HIV risk reduction counselling.

### Adherence Assessment

Adherence in this study was measured via four methods: EM with the medication event monitoring system (MEMS, WestRock, Switzerland); self-reported taking of pills; and drug concentrations of tenofovir (TFV) in plasma and hair. EM data were downloaded from the MEMS at monthly study visits, and staff-related openings were removed from the dataset. Self-reported pill taking was assessed using a follow-back calendar method [15–17] over the time since the prior study visit. At clinic visits 8 and 16, the study staff and participant reviewed the memory aid completed by the participant in the last 28 days. The memory aid data were used to complete the calendar with the staff helping the participant to recall each day’s activity where necessary. MEMS opening and self-report were capped at one dose per day when there was more than one opening or self-reported pill-taking, respectively, on the same day. Plasma samples were collected every 4 weeks, with hair sampling at 8 and 16 weeks. The procedures on sample handling and testing for the PK methods are reported elsewhere [8]. The lower limits of detection for each PK measure were as follows: plasma TFV concentration 0.31 ng/ml and hair TFV concentration 0.002 ng/mg [8].

### Statistical Analysis

We limited our analysis to participants receiving daily active drug in the IAVI trial and summarised participant characteristics by counts and percentages. Data from Kenya were further limited to that from MSM participants because only five FSW (in whom behaviour and/or PK parameters may have differed from the MSM) participated. Missing data were excluded from analysis (i.e., no assumptions or imputations were performed). We aligned measures over equivalent dosing windows. For example, we used data for the 7 days prior to weeks 8 and 16 to compare plasma drug levels with EM and self-reported adherence. For comparisons with hair drug concentration, we used EM and self-reported adherence as recorded or reported over the 28 days prior to weeks 8 and 16 (averaged by week).

We compared adherence measurements obtained at the 8- and 16-week study visits. Analyses were stratified by study site, given socio-behavioral differences in the Kenyan and Ugandan populations, and higher adherence to study product in Uganda compared to Kenya as determined by five adherence measures in prior analyses [13, 14]. First, we categorised each of the adherence measures assessed in this study as low, moderate, and high, as shown in Table 1. The PK categorisations were based on the median values of each adherence measure from previous directly-observed therapy (DOT) studies, where TDF was administered to HIV-negative volunteers in a variety of dosing patterns to establish “adherence benchmarks” in the relevant biological matrix, for hair [18] or plasma [19]. These cut-offs have been shown to be associated with clinical outcomes such as toxicities or seroconversion in other PrEP studies [20–24]. Medians were chosen over other potential thresholds (e.g., top tertile) because of the relatively small sample size, which can exaggerate the effect of outliers, and a high degree of inter- and intra-individual variability in drug metabolism. We estimated categorical concordance when both measures were matched (i.e., high–high, moderate–moderate, or low–low), expressed as a percentage. Additionally, Pearson correlation coefficients were calculated to determine correlations between the different adherence measures using continuous values. For EM and self-report measures, the actual number of doses taken represented by self-report or MEMS opening were used for correlations.

**Table 1:** Adherence categories for each of the five adherence metrics

Adherence level	MEMS	Self-reported doses per week	Plasma TFV-DP (ng/ml)	Hair TFV-DP (ng/mg)
Low	0– < 29%	0–2	≤ 5.9	≤ 0.012
Moderate	29– < 71%	3–5	> 5.9– < 52.2	> 0.012– < 0.038
High	71–100%	6–7	52.2 +	0.038 +

*MEMS* medication event monitoring system, *TFV* tenofovir, *TFV*-*DP* tenofovir diphosphate

To assess the incremental value of multiple adherence measures when used in combination, we compared distinct categories of EM adherence with the other adherence measures. We used a discriminant (C-statistic) analysis for single or combined methods for adherence measurement via logistic regression models with the outcome as binary (Table 1 moderate was combined with low and compared to high) and displayed by receiver operating characteristic (ROC) curve area. Data from both sites were combined due to sample size limitations. We also used univariate and multivariate linear models to show percentage variability (using models R-squared) between EM (continuous variable) and other adherence measures, either individually or in combination, taking in consideration repeated measures. While the accuracy of EM is potentially limited by pocket dosing (i.e., removal of multiple pills with one bottle opening) and curiosity openings (i.e., opening the bottle without removing pills), this measure has been shown to provide accurate estimates of day-to-day adherence behaviour [25, 26]. Therefore, EM was chosen as the comparison (standard) in this analysis. Drug concentration levels were log-transformed for use in linear regression models. All PK concentrations in this study that were below the detection limit of each assay were set as equal to that limit, and the detection limit was added to all concentrations prior to log transformation. A separate analysis based on hair as the comparison (standard) as opposed to EM was performed.

## Results

### Participant Characteristics

**Fig. 1:**
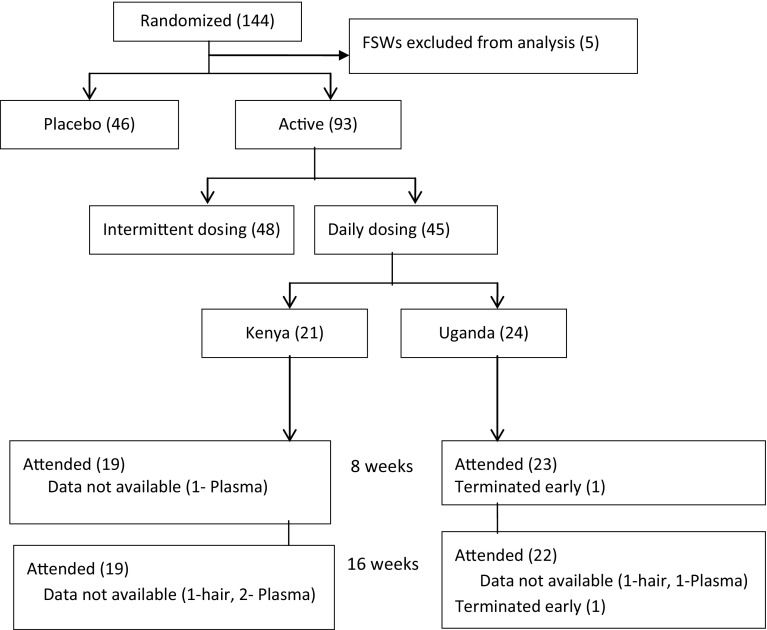
Study profile on randomization and follow up of participants at both Uganda and Kenya sites

Figure 1 shows the number of participants randomised, those missing PK samples at a given visit, and those terminating the study early. Of the 139 participants randomized in all sites (Uganda and Kenya) of the IAVI trial, 45 (32.4%) were assigned to daily active tenofovir/emtricitabine and were therefore eligible for this analysis [Uganda (N = 24) and Kenya (N = 21)]. Of these, 66.7% were male with a mean age of 29.2 years (standard deviation [SD] 6.9).

### Adherence Adjudicated by Each Measure by Time Frame

Median adherence in Ugandan participants was high by EM [7 openings per week, interquartile range (IQR) 6, 7], self-report (7 doses per week; IQR 7, 7), plasma (70.5 ng/ml; IQR 38.9, 94.6), and hair (0.07 ng/mg; IQR 0.05, 0.11). In Kenyan participants, median (IQR) adherence was as follows: moderate by EM [5 openings per week, interquartile range (IQR) 4, 7] and high by self-report (7 doses per week; IQR 6, 7), plasma (81.0 ng/ml; IQR 40.0, 148.2) and hair (0.07 ng/mg; IQR 0.03, 0.08).

### Concordance Among Adherence Measures

As shown in Table 2, agreement was moderate to high with percent concordance of 63.6–93.3% among all measures at the Ugandan site. Agreement among measures at the Kenyan site was generally lower than in Uganda, ranging from 28.1–71.9%. Table 2 also indicates the direction of the discordance (i.e., higher or lower for each adherence measure compared to the others).

**Table 2:** Concordance and correlations (Pearson’s coefficients) between non-PK and PK adherence measures among volunteers in Uganda (bold) and Kenya (italics)

Methods	Agreement	EM		Self-report		Plasma		Hair	
		CC	N (%)	CC	N (%)	CC	N (%)	CC	N (%)
EM, N = 45	Concordant	1.0	1.0	*0.02*	*9 (60.0)*	0.68**	*22 (68.8)*	0.85**	*23 (71.9)*
	Discordant (EM > other)				*4 (26.7)*		*6 (18.7)*		*5 (15.6)*
	Discordant (EM < other)				*2 (13.3)*		*4 (12.5)*		*4 (15.8)*
Self-report, N = 21	Concordant	**0.02**	**42 (93.3)**	1.0	1.0	− 0.43**	*7 (46.7)*	*− 0.20*	*9 (28.1)*
	Discordant (SR > other)		**2 (4.4)**				*3 (20.0)*		*8 (25.0)*
	Discordant (SR < other)		**1 (2.2)**				*5 (33.3)*		*15 (46.9)*
Plasma, N = 36	Concordant	**0.20**	**31 (70.4)**	− **0.08**	**28 (63.6)**	1.0	1.0	0.49**	*18 (56.3)*
	Discordant (plasma > other)		**13 (29.6)**		**1 (2.3)**				*7 (21.9)*
	Discordant (plasma < other)		**0 (0.0)**		**15 (34.1)**				*7 (21.9)*
Hair, N = 42	Concordant	**0.41** **	**36 (81.8)**	− **0.01**	**37 (84.0)**	**0.29**	**31 (72.1)**	1.0	1.0
	Discordant (hair < other)		**6 (13.6)**		**1 (2.3)**		**3 (7.0)**		
	Discordant (hair < other)		**2 (4.6)**		**6 (13.7)**		**9 (20.9)**		

Data reflect measurements at 8 and 16 weeks of follow-up for a total of 45 assessments in Uganda and 39 in Kenya

*EM* electronic monitoring, *CC* correlation coefficient

**Statistically significant p < 0.05

### Correlations Among Adherence Measures

In general, hair drug concentrations were most highly correlated with EM adherence with coefficients of 0.41 in Uganda and 0.85 in Kenya (all p < 0.01). The correlation for plasma drug concentrations with EM adherence was similarly high for the Kenyan site (coefficient 0.68, p < 0.01), but lower for the Ugandan site (coefficient 0.20, p = 0.19). The correlations between self-reported adherence and PK measures were poor at both sites with coefficients of − 0.43 to − 0.01 (all p > 0.05). The correlations between hair and the plasma measures were moderate, ranging from 0.29 to 0.58 for both sites (p = 0.06, p < 0.01).

### Incremental Value of Multiple Adherence Measures in Combination Compared to Electronic Monitoring

**Fig. 2:**
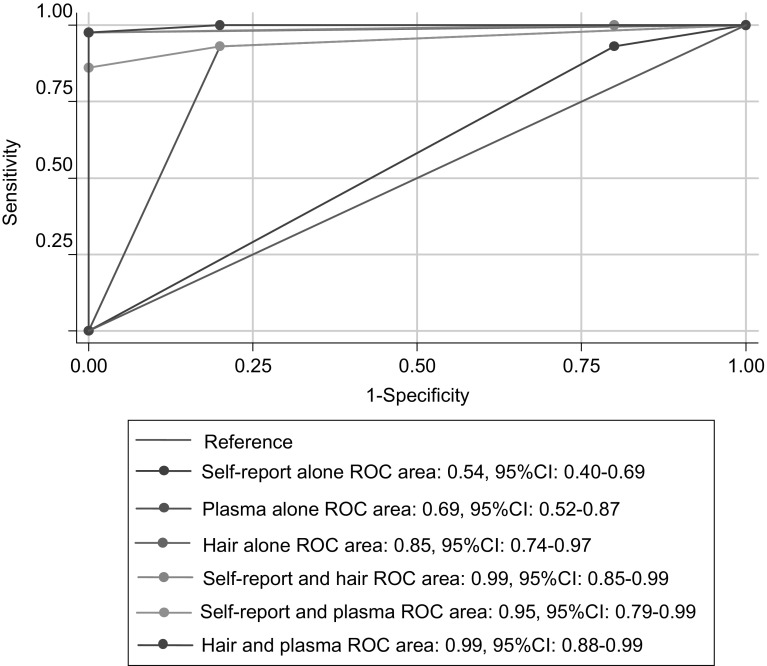
Maximal discrimination between high and moderate or low EM adherence based on a single alternate adherence measure or combinations of adherence measures

Figure 2 and Table 3 indicate the ability of each adherence measure individually or in combination to discriminate among high versus moderate or low EM adherence. Discrimination was poor for the self-reported measure alone [area under receiver operating curve (AROC) 0.54, 95% CI 0.40–0.69] and best for the hair measure alone, AROC 0.85, 95% CI 0.74–0.97. When combining two measures, discrimination improved with an AROC of > 0.9 for all combinations. The AROC was highest for a combination of self-report, hair, and plasma at an AROC = 0.999, leading to the optimal ROC curve for all possible combinations of measures (Fig. 2).

**Table 3:** Ability of each adherence measure individually or in combination to discriminate among low, moderate, and high electronic monitoring adherence

Measure	Area under the curve (AROC) 95% CI
Single measure	
Self-report	0.54 (0.40–0.69)
Plasma	0.69 (0.52–0.87)
Hair	0.85 (0.74–0.97)
Combination of two measures	
Self-report + hair	0.99 (0.85–0.99)
Plasma + hair	0.99 (0.88–0.99)
Self-report + plasma	0.95 (0.79–0.99)
Combination of three measures	
Self-report + plasma + hair	0.99 (0.92–0.99)

Values indicate the C-statistic (area under the curve). Data from both sites were combined due to sample size limitations

We determined the percent variability in EM adherence explained by each of the other adherence measures individually or in combination. At both sites, hair as a single adherence measure performed the best; hair explained 17.0 and 41.0% of variability in EM adherence in Uganda and Kenya, respectively. In assessing combinations of two adherence measures, the percent of explained variability improved and was highest for hair plus self-report in Uganda (17.4%) and hair plus plasma in Kenya (61.4%) Table 4. A combination of three measures (self-report, hair and plasma) did not provide a sizeable improvement compared to the highest shown by a combination of two measures [Uganda (20.0%) and Kenya (63.5%)]. Overall, the percent of variability in EM adherence explained by other adherence measures was lower in Uganda compared to Kenya.

**Table 4:** Electronic monitoring adherence compared to each of the other adherence measures individually or in combination (adjusting for different measures) using univariate and multivariate linear models

Measures	Prediction of EM adherence	
	Uganda	Kenya
	Adjusted R	Adjusted R
Single measure		
Self-report	0.0	0.0
Plasma	3.9	41.9
Hair	17.0	41.0
Combination of two measures		
Self-report + plasma	4.1	53.0
Self-report + hair	17.4	40.6
Plasma + hair	16.2	61.4
Combination of three measures		
Self-report + plasma + hair	20.0	63.5

In the separate analysis using hair as the standard comparison, similar patterns were observed as to when EM was used as the standard comparison). SR adherence performed poorly [(AROC) 0.52, 95% CI 0.41–0.64 for discrimination; 1.0 variability explained], whereas SR plus EM performed best [(AROC) 0.83, 95% CI 0.72–0.93 for discrimination; 53.8 variability explained].

## Discussion

Among participants taking daily PrEP in a clinical trial, we described patterns of adherence to PrEP by combining short and long-term pharmacokinetic measures and aligning them with concurrent electronic data monitoring metrics and self-reported adherence. We found evidence of consistently high adherence over different durations of time as assessed by multiple measures at both sites. In Kenya, the EM measure showed more moderate levels of adherence than in Uganda. Prior qualitative work with this population found that challenges, such as complexities of daily life, may have contributed to lower adherence for MSM [27].

We found mixed results in the concordance and correlation analyses. Concordance was high in comparisons of self-report, EM, and plasma and hair drug levels at the Ugandan site, although more moderate concordance was seen among these measures at the Kenyan site. Interestingly, the correlations between measures were higher in the Kenyan population compared to the Ugandan population, although correlations were generally lower than concordance. This finding may reflect the distribution of the data and the manner in which we created the PK categories for low, moderate, and high adherence. Our choice to use medians makes sense for this small study and the need to avoid the influence of outliers. However, values that fall just on either side of the medians may be misclassified, thus causing differences in analyses of concordance versus correlation. Furthermore, while the correlations are determined using data from these trials, the categorizations for concordance determination were based on median values from previous studies.

Importantly, limitations in the various adherence measures may have affected the accuracy of our comparisons. For instance, among the MSM at the Kenyan site, stigma [27] may have resulted in a reduced desire to use the EM device. Participants may have truly adhered to their medication through pocket dosing (e.g., opening the device and removing multiple pills on 1 day), thus resulting in high drug levels in plasma and hair, and decreased concordance of EM with other measures. Indeed, much of the discordance in this population reflected higher adherence as assessed by the PK measures and lower adherence as adjudicated by EM. Social desirability may have led to inaccuracies in self-reported adherence, as has been seen in multiple prior studies [25, 28]. Additionally, drug concentration determinations may have been limited by variation in sample processing, differences in metabolism within and between individuals, drug–drug interactions, and/or variable dose timing relative to sample collection (relevant only for plasma samples, in which the half-life of tenofovir is approximately 17 h) [8, 10, 19].

A ROC analysis was used to determine the incremental value of combining multiple adherence measures in order to discriminate among low, moderate, and high EM adherence. As expected, we found that self-report alone performed poorly (AROC 0.54). This finding is similar to an analysis in the Ancillary Adherence Study within the Partners PrEP Study where discrimination of steady-state daily dosing versus less than steady state dosing for plasma tenofovir was poor for self-report (AROC 0.52). Our results indicate that hair drug levels offered the best discriminant ability (AROC 0.85) as a single PK measure. This finding suggests that a long-term measure of adherence, as represented by hair levels, can help summarize the day-to-day variability captured by the EM device. Using one PK measure plus self-report improved the discriminant ability to about 90% and a combination of two PK metrics and self-report predicted the EM data even further. Differences in this analysis may exist by site, but the small sample size prevented site stratification.

In the regression analyses to predict variability in the EM adherence measure, hair drug levels explained the most variability at both sites, although plasma levels performed nearly as well in Kenya. The combination of hair and plasma drug concentrations explained nearly as much of the variability as combinations of three measures. This finding argues for combining short-term (e.g., plasma) and long-term (e.g., hair) measures to obtain a comprehensive picture of adherence over time. The incremental value of the other adherence measures either singularly or in combination was higher in the Kenya site than the Uganda site. Reasons for this finding are unclear, but could be related to the higher correlations between measures at the Kenya site. Both approaches showed generally increasing ability to discriminate adherence patterns with an increasing number of measures. However, the choice for the number of adherence measures used in a given study is dependent on resources and competing scientific priorities. Multiple measures may be most appropriate when an accurate assessment of adherence is needed for assessing biologic efficacy and/or triggering interventions [29]. If only one measure can be employed, our analysis suggests that either hair levels or EM monitoring can be used, with the former providing an overall measure adherence and the latter indicating patterns.

The major strength of this analysis includes the inclusion of multiple adherence methods, including concurrent collection of both non-PK and PK measures. This data provide a rare opportunity for aligning the adherence measures over the same time period. Although there were differences in the comparisons among measures by site, the data taken as a whole show that correlations were highest between the EM measure and each of the PK measures, with hair levels exhibiting the best performance.

There are important limitations with this analysis. First, because we were interested in only the active arm of the two studies and the daily dosing strategy, the sample size was small and in some instance the data analyses could not be stratified by site. The small sample size limits us making conclusive assertions about the hypothesised results. Therefore, the results of this analysis need to be interpreted with caution. Second, the sample was predominantly men, which could limit extrapolation of results to populations dominated by women. Third, we chose to use EM as the comparison standard for this analysis, as it has been previously shown to be an excellent measure for day-to-day adherence behaviour [25, 26]. It has also been shown to meaningfully discriminate between high rates of daily dosing compared to low rates [8]. Similar analyses could also be pursued to assess the ability to discriminate adherence per the PK measures. Finally, further investigations on PrEP adherence need to be carried out in routine care settings because adherence patterns in a placebo-controlled trial environment could differ from the real-world setting where participants are taking a product already known to work.

In sum, this analysis indicates that objective measures of adherence are more informative than self-report and that employing multiple measures of adherence is likely to increase accuracy in estimating adherence behaviour. Specifically, the combination of adherence measures that reveal behaviour over the long-term (hair) plus another short-term measure (plasma) may be useful to assess patterns of drug-taking. Important differences in measure performance, however, may arise in different populations and must be considered carefully for any study, most feasibly in the planning stage. Hair appears to be a good choice to include as an adherence measure where limited testing is available. Drug levels in dried blood spots also assess longer-term adherence [30], but were not collected in this particular study. Given the importance of adherence in achieving effective protection against HIV with PrEP, these data provide valuable insights in the design of future prevention studies.
